# Characterization of Biostimulant Mode of Action Using Novel Multi-Trait High-Throughput Screening of *Arabidopsis* Germination and Rosette Growth

**DOI:** 10.3389/fpls.2018.01327

**Published:** 2018-09-13

**Authors:** Lydia Ugena, Adéla Hýlová, Kateřina Podlešáková, Jan F. Humplík, Karel Doležal, Nuria De Diego, Lukáš Spíchal

**Affiliations:** ^1^Department of Chemical Biology and Genetics, Centre of the Region Haná for Biotechnological and Agricultural Research, Faculty of Science, Palacký University, Olomouc, Czechia; ^2^Laboratory of Growth Regulators, Centre of the Region Haná for Biotechnological and Agricultural Research, Institute of Experimental Botany, Czech Academy of Sciences, Olomouc, Czechia

**Keywords:** biostimulants, multi-trait high-throughput screening assay, proline, polyamines, plant biostimulant characterization index, salinity

## Abstract

Environmental stresses have a significant effect on agricultural crop productivity worldwide. Exposure of seeds to abiotic stresses, such as salinity among others, results in lower seed viability, reduced germination, and poor seedling establishment. Alternative agronomic practices, e.g., the use of plant biostimulants, have attracted considerable interest from the scientific community and commercial enterprises. Biostimulants, i.e., products of biological origin (including bacteria, fungi, seaweeds, higher plants, or animals) have significant potential for (i) improving physiological processes in plants and (ii) stimulating germination, growth and stress tolerance. However, biostimulants are diverse, and can range from single compounds to complex matrices with different groups of bioactive components that have only been partly characterized. Due to the complex mixtures of biologically active compounds present in biostimulants, efficient methods for characterizing their potential mode of action are needed. In this study, we report the development of a novel complex approach to biological activity testing, based on multi-trait high-throughput screening (MTHTS) of *Arabidopsis* characteristics. These include the *in vitro* germination rate, early seedling establishment capacity, growth capacity under stress and stress response. The method is suitable for identifying new biostimulants and characterizing their mode of action. Representatives of compatible solutes such as amino acids and polyamines known to be present in many of the biostimulant irrespective of their origin, i.e., well-established biostimulants that enhance stress tolerance and crop productivity, were used for the assay optimization and validation. The selected compounds were applied through seed priming over a broad concentration range and the effect was investigated simultaneously under control, moderate stress and severe salt stress conditions. The new MTHTS approach represents a powerful tool in the field of biostimulant research and development and offers direct classification of the biostimulants mode of action into three categories: (1) plant growth promotors/inhibitors, (2) stress alleviators, and (3) combined action.

## Introduction

Agricultural crop production will be extremely challenging in the coming decades. Due to the increase in population, a 50% (maximum) increase in the demand for food is expected by 2030. During the growing season, crops around the world are subjected to environmental stresses that affect plant germination, metabolism, growth and yield. Breeders worldwide have therefore focused on quantitative analyses of plant traits in order to accelerate the development of appropriate strategies for improving lines or varieties which are adaptable to resource-limited environments (Rahaman et al., 2017). Soil salinity is an important environmental factor that results in decreased crop productivity on a global scale. In fact, owing to this factor, an estimated 1.5 million hectares of land is taken out of production each year and by 2050 a 50% loss of cultivable lands is expected (Ibrahim, 2016).

The application of biostimulants represents one of the most innovative and promising strategies for minimizing stress impact, including salinity. A plant biostimulant is defined as a material of biological origin which includes bacteria, fungi, seaweeds, higher plants, animals and humate-containing raw materials (Sharma et al., 2014; Yakhin et al., 2016; Cristiano et al., 2018). This material induces beneficial plant processes (including nutrient uptake, nutrient use efficiency, tolerance to abiotic stress and crop quality), independently of its nutrient content (Calvo et al., 2014; Yakhin et al., 2016). Exposure of seeds to abiotic stresses, such as salinity among others, results in lower seed viability, reduced germination, and poor seedling establishment (Savvides et al., 2016). Increasing the salt concentration of the soil leads to a decrease in the germination percentage and delays the germination starting point (Kaveh et al., 2011; Thiam et al., 2013; Ibrahim, 2016). Seed-priming might improve seed stress-tolerance through ‘priming memory,’ which is established during priming and can be recruited later when seeds are exposed to stresses during germination (Chen and Arora, 2013). Seeds primed with biostimulants from varied origins trigger fast seed germination (Zeng et al., 2012; Colla et al., 2014; Garcia-Gonzalez and Sommerfeld, 2016). Besides, priming seeds with certain biostimulants can promote tolerance to adverse environmental conditions during the imbibition and germination stages (Mahdavi, 2013; Sharma et al., 2014; Pichyangkura and Chadchawan, 2015; Van Oosten et al., 2017).

Recently, the global biostimulant market has grown rapidly and, to satisfy crop requirements, many companies are actively introducing various innovative products and ingredients (Calvo et al., 2014; Sharma et al., 2014). However, in general, the raw materials used by the biostimulant manufacturers exhibit considerable compositional variations which may impact on the composition and concentration of major components (Povero et al., 2016; Sharma et al., 2016). The origin of biostimulants is diverse, and can range from single compounds to complex matrices with different groups of bioactive components that have only been partly characterized (du Jardin, 2015). Irrespective of their complexity, biostimulants are known to contain different groups of plant signaling compounds such as plant hormones, amino acids, and polyamines among others (Craigie, 2011; du Jardin, 2015). The exogenous application of these signaling molecules has been reported to ameliorate the adverse effect of stress through a sophisticated crosstalk among them leading to the activation of conserved pathways [reviewed in Podlešáková et al. (2018)].

In this work we present a novel approach for biostimulant mode of action characterization based on multi-trait high-throughput screening (MTHTS) of *Arabidopsis* germination and rosette growth under salinity. The analyzed traits included the germination rate, rosette growth rate and color. The potential of the approach was demonstrated by applying (via seed priming) representatives of the most common compounds present in biostimulants (i.e., polyamines and amino acids). In addition, we optimized the principles of two previously described protocols for implementation into the MTHTS approach. These included (i) the fast scoring of the germination rate based on a standardized 96-well plate test coupled with spectrophotometric reading of tetrazolium salt reduction (Pouvreau et al., 2013) and (ii) an automated method for high-throughput screening of *Arabidopsis* rosette growth in multi-well plates (De Diego et al., 2017). A highly efficient and reliable method for characterizing biostimulant efficacy at various salt stress levels was realized by developing and combining a high-throughput seed germination assay in *Arabidopsis* with the improved *Arabidopsis* rosette growth assay.

## Materials and Methods

### HTS of *Arabidopsis* *in vitro* Seed Germination

*Arabidopsis thaliana* (L.) Heynh seeds (accession Col-0) were surface-sterilized by soaking in 70% Ethanol plus 0.01% Triton X-100 for 10 min. After that, the seeds were washed with sterilized water and then resuspended at a density of 10 g L^−1^ in 1 mM HEPES [4-(2-hydroxyethyl)-1-piperazineethanesulfonic acid] buffer (Carl Roth GmbH + Co. KG., Germany) (pH 7.5). Seeds were stratified at 4°C in the dark for 72 h. To investigate the effect of biostimulants on *Arabidopsis* *in vitro* seed germination, four single active compounds commonly present in many commercial biostimulant products were selected for seed priming; three polyamines: putrescine (Put) (1,4-butanediamine dihydrochloride), spermidine (Spd) (*N*-(3-aminopropyl)-1,4-butanediamine trihydrochloride), spermine (Spm) [N-(3-Aminopropyl)-1,4-butanediamine trihydrochloride] and the amino acid *L*-proline (Pro) [(*S*)-Pyrrolidine-2-carboxylic acid], all purchased from Sigma-Aldrich, Inc., (Germany). These compounds were added before the stratification, reaching final concentrations of 0.001, 0.01, 0.1, or 1 mM. After the cold stratification, seed suspension was washed three times with 20 mL sterile water to remove the biostimulants. In the last wash half of the water volume was removed and an additional 10 mL solution of sterilized 0.1% agarose with 1 mM of HEPES buffer was added. This is because seeds do not sediment in 0.05% agarose and are suspended in an adequate homogeneous solution for pipetting. The 96-well plate was filled with the seed suspension, 50 μL per well, representing ∼20–30 seeds per well. The final volume was adjusted to 100 μL per well with demineralized water or, in the case of the salt stress treatments, a NaCl solution that yields a final concentration of 75 or 150 mM NaCl in the well. Plates were sealed and incubated for seed germination at 21°C in darkness.

**FIGURE 1:**
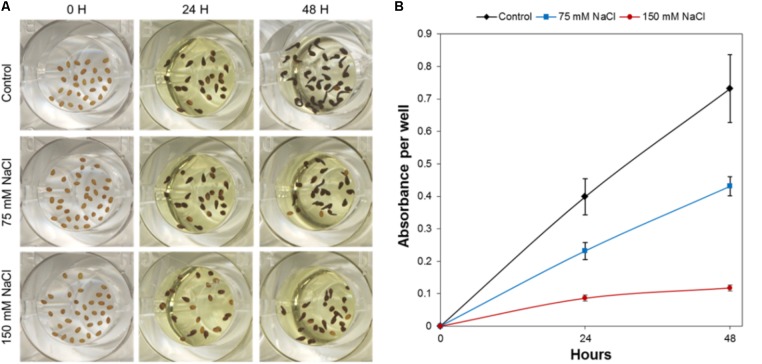
HTS of *Arabidopsis* germination under control, moderate (75 mM NaCl), and severe (150 mM NaCl) stress conditions. **(A)** Characteristics of *Arabidopsis* seeds in one well of the 96-well plate before germination (0 h), and after 24 and 48 h, respectively, of germination with subsequent MTT treatment. **(B)** Absorbance of MTT after solubilization of formazan from *Arabidopsis* seeds germinated under control, moderate (75 mM NaCl), and severe (150 mM NaCl) stress conditions. The values represent *Mean ± SE*.

For the quantification of the *Arabidopsis* germination rate, the methylthiazolyldiphenyl-tetrazolium bromide (MTT; Sigma-Aldrich, Inc.) assay was performed in accordance with Pouvreau et al. (2013). In this process, 10 μL of 0.5% MTT solution per well was added after 24 or 48 h under germination conditions. Plates were placed in the culture chamber for an additional 24 h in darkness and a redox reaction, which is a reduction of MTT to formazan, lasted for 24 h (**Figure 1**). After MTT addition, the formazan salt deposit was solubilized by adding 100 μL of lysis buffer (10% Triton X-100, 0.04 mol L^−1^ HCl in isopropanol) to each well, and holding at 21°C in darkness for another 24 h. Subsequently, the absorbance was read with a BioTek^TM^ Synergy^TM^ H4 Hybrid Microplate Reader (BioTek Instruments, Inc., United States). For each well, the final absorbance was calculated by subtracting the absorbance at a reference wavelength of 690 nm from a test absorbance of 570 nm (A570–690 nm).

#### Image Acquisition and Data Analysis

Images were acquired by scanning each plate twice (HP ScanJet 5300c; resolution 1200 DPI; HP Development Company, L.P., United States), immediately after placing the seeds in the 96 multi-well plates (0 h) and after 24 or 48 h under seed-germination conditions with the subsequent 24 h MTT treatment. The images were saved as TIFF format. For seed counting, the images of *Arabidopsis* seeds at 0 h (immediately after cold stratification) were used and the number of seeds per well was estimated using an in-house software routine implemented in MATLAB R2015. The free of charge access to the software application for academical purposes is described in the next section.

### HTS of *Arabidopsis* Rosette Growth

#### Experimental Setup and Assay Conditions

The protocol for analysis of *Arabidopsis* rosette growth described by De Diego et al. (2017) was modified as follows. Seeds of *A. thaliana* (ecotype Col-0) were surface- sterilized and sown on 12 cm × 12 cm square plates containing a 0.5× MS medium (Murashige and Skoog, 1962) (pH 5.7) supplemented with a gelling agent (0.6% Phytagel; Sigma-Aldrich, Germany). The seeds were kept for 4 days at 4°C in the dark (in the case of primed variants, the growth medium contained the tested biostimulant described below). The plates were then positioned vertically in a growth chamber under controlled conditions (22°C, 16/8 h light/dark cycle with the light cycle starting at 5 a.m., photon irradiance: 120 μmol photons of PAR m^−2^ s^−1^). Three days after germination, seedlings of similar size were transferred under sterile conditions into 48-well plates (Jetbiofil, Guangzhou, China). One seedling was transferred to each well filled with 850 μL 1× MS medium (pH 5.7; supplemented with 0.6% Phytagel), with NaCl added for different salt stress intensities (75 and 150 mM NaCl) and the plates were sealed with perforated transparent foil allowing gas and water exchange. The 48-well plates containing the transferred *Arabidopsis* seedlings were placed the OloPhen platform^1^ that uses the PlantScreen^TM^ XYZ system installed in a growth chamber with a controlled environment and cool-white LED and far-red LED lighting (Photon Systems Instruments, Brno, Czechia). The conditions were set to simulate a long day with a regime of at 22°C/20°C in a 16/8 h light/dark cycle, an irradiance of 120 mmol photons of PAR m^−2^ s^−1^ and a relative humidity of 60%. The PlantScreen^TM^ XYZ system consists of a robotically driven arm holding an RGB camera with customized lighting panel and growing tables with a total area of approximately 7 m^2^. To increase the throughput of the assay, the capacity of the growing area was improved to accommodate in total 572 multi-well. The XYZ robotic arm was automatically moved above the plates to take RGB images of single plates from the top. The imaging of each 48 well plate was performed twice per day (at 10 a.m. and 4 p.m.) for 7 days. RGB images (resolution 2500 × 2000 pixels) of a single plate with a file size of approximately 10 MB in the PNG compression format were stored in a database on a server, using a filename containing information about the acquisition time and the (x, y) coordinates of the camera. The data were automatically stored in PlantScreen XYZ database, exported by PlantScreen Data Analyzer software and analyzed using an in-house software routine implemented in MATLAB R2015.

The software application for *Arabidopsis* rosette growth analysis (same as for above described *Arabidopsis* seed counting) can be used without any charge upon obtaining a license from the author. The license can be obtained by e-mail to Palacky University upon agreeing not to use the application for commercial purpose. After obtaining the license, the enduser will be provided (free of charge) with the MCRInstaller.exe. MCRInstaller simulates the MATLAB environment on computers where MATLAB is not installed and enables to execute the applications. To obtain the application executable files, please contact the author Tomas Furst by email tomas.furst@upol.cz. The email must contain the following statement: “Neither the application nor the MCRInstaller will be used for any commercial purpose.”

#### Seed Priming With Biostimulants

The biostimulant effect was determined using Put, Spd, Spm, and Pro for seed priming. After sterilization, the aforementioned seeds were placed on 12 cm × 12 cm square plates containing a 0.5× MS medium (pH 5.7) supplemented with the tested compounds at four concentrations (0.001, 0.01, 0.1, or 1 mM). After 4 days in the dark and 3 days of germination, seedlings were transferred into 48 multi-well plates filled with a 1× MS with/without salt (75 or 150 mM NaCl solution) addition. Two plates per growth condition, compound and concentration (96 seedlings) were used as replicates for the control and 75 mM NaCl. Due to the high mortality of seedlings under severe salt stress conditions, three plates for the seedling in 150 mM NaCl were used to obtain sufficient reproducible data and an adequate number of measurable individuals.

#### Biometrical Parameters

The changes in green area (Pixels) were measured twice per day in each *Arabidopsis* seedling using the aforementioned automatic system. From the obtained data, the relative growth rate (RGR) per hour or day was estimated for each replicate and variant as follows:

(1) $$\text{RGR}=[\ln{\left(\text{green area}\right)}_{\text{t}i}-\ln{\left(\text{green area}\right)}_{\text{t}i-1}]/(\text{t}i-\text{t}i-1)$$

Where t*i* is the *i* time (h or days).

#### Determination of the Leaf Color in *Arabidopsis* Rosette Under Control and Salt Stress Conditions

For non-invasive estimation of the changes in leaf color, we calculated three vegetative indices (NGRDI, GLI, and VARI) which have exhibited correlation with the plant biomass, nutrient status or tolerance to abiotic stress (Gitelson et al., 2002; Perry and Roberts, 2008; Hunt et al., 2013). The images captured on the seventh day of an *Arabidopsis* rosette growth assay subjected to HTS were segmented for the extraction of leaf rosettes using software described in our previous report (De Diego et al., 2017). Afterward, the values corresponding to particular color channels (red = R, green = G, and blue = B) were extracted for each pixel within the plant mask, and the vegetative indices were calculated as follows:Normalized green red difference index

(2) NGRDI−(G-R)/(G+R)

Green leaf index

(3) GLI=(2G-R-B)/(2G+R+B)

Visible atmospherically resistant index

(4) VARI=(G-R)/(G+R−B)

Subsequently, indices representing particular seedlings were determined by calculating the mean values for each plant mask. The mean value for each 48-well plate was then calculated.

### Statistical Analysis

The one-way analysis of variance (ANOVA) was used to assess the differences between the projected areas (Pixels) or seed germination (absorbance) of two or more plant groups at a particular time-point. The test compares the variance (or variation) between the data samples to variation within each particular sample. When ANOVA was significant the differences among groups was determined using Dunn & Sidák’s approach.

The relationship among traits was analyzed via Pearson’s correlation. Furthermore, the significance of the regression was determined by applying a Student’s *t*-test to the linear curves and after linearization of non-linear curves.

## Results

### Development of HTS of *Arabidopsis* Seed Germination Under Control and Salt Stress Conditions

**FIGURE 2:**
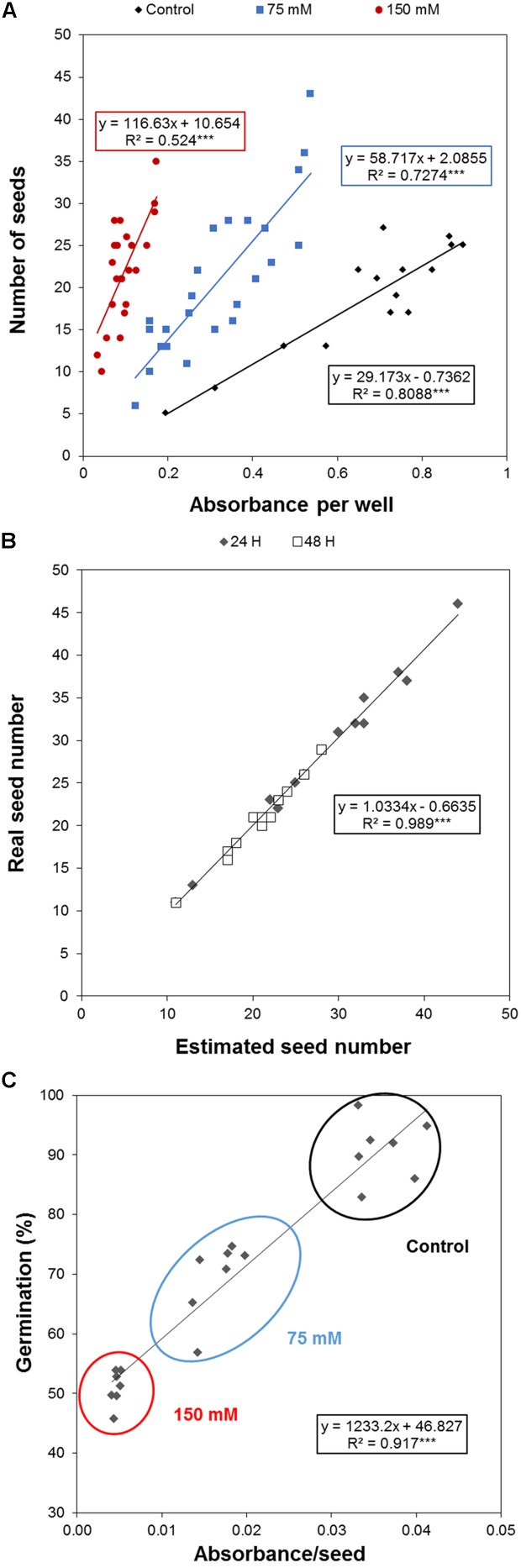
Optimization of *Arabidopsis* germination HTS. Correlation between the **(A)** number of seeds per well and the absorbance under control, moderate (75 mM NaCl), and severe (150 mM NaCl) stress conditions, **(B)** manually counted number of seeds and the software-estimated number of seeds per well, and **(C)** germination rate (%) and the estimated number of seeds. The equation of the curve and the Pearson’s correlation coefficient with significance according to ANOVA were calculated. ^∗∗∗^*p* < 0.001.

To efficiently determine the effect of biostimulant priming on the seed germination rate, we developed a HTS assay for seed germination using the MTT method proposed by Pouvreau et al. (2013). In this method, the MTT is used as a marker of metabolic activity in the embryo and its reduction to purple formazan can be quantified spectrophotometrically in a microtiter plate. We optimized this assay for *Arabidopsis* seeds and validated the assay for determining the effect of salinity at two time points (i.e., 24 and 48 h; see **Figure 1**). The severity of the salinity was expected to exert a dose-dependent negative effect on seed germination (seed staining and radicle length decrease; **Figure 1A**), leading to a decrease in the absorbance values measured (**Figure 1B**). During optimization of the assay, we observed a strong correlation between the absorbance values and the number of seeds per well under all three growth conditions (**Figure 2A**). Thus, a stable number of seeds per well was critical to reducing the variability in the experiment. This is, however, technically difficult under HTS conditions when a high number of wells/plates must be rapidly filled. To solve this problem we used 0.05% agarose solution allowing homogeneous suspension of seeds through vortexing. This way using multi-step pipette the average number of 21 ± 5.4 (mean ± SD) seeds per well was achieved. Besides, we handled the relatively high variability (∼25%) by developing an automatic simple software that counts the exact number of seeds per well (rather than finding a technical solution that allows precise and repeated filling of the plate wells with the same number of seeds). Using this software routine, the measured absorbance per well can be recounted to the absorbance per seed. In the first step of this process, the software identifies the wells in the plate. The seeds are then identified via simple thresholding in the R, G, and B channels and single seeds or clusters of seeds are subsequently separated from the background. Afterward, single seeds are distinguished from clusters by computing the solidity (i.e., the ratio of the area of the convex hull of an object to the area of an object) of each object. Single seeds have a high solidity (usually >0.9), whereas clusters of seeds are larger and have lower solidity. The number of seeds in a cluster is estimated by dividing the area of the cluster by the average seed area which is determined from previous runs of the software. The accuracy of the software was determined by manually counting the seeds on several plates, and a high correlation was obtained between the real number and the software-estimated number of seeds (**Figure 2B**). As shown in **Table 1**, the counting of the seeds allowed us to reduce the dispersion of the absorbance per variant, with an at least three times lower standard deviation (28 vs. 9%) in the two analyzed points at 24 and 48 h. Thus, we observed a significant correlation (*p* < 0001) between the absorbance per seed and the percentage of *Arabidopsis* seeds germinated under control and salt stress conditions (**Figure 2C**).

**Table 1:** Comparison of measured overall formazan absorbance and the absorbance recounted per seed after 24 or 48 h of germination.

	24 h		48 h	
	Absorbance	Absorbance/seed	Absorbance	Absorbance/seed
Mean	0.399	0.019	0.73	0.041
SD	0.112	0.002	0.21	0.003
%	28.08	9.14	28.52	8.28

*Mean ± SD and contribution of the SD to each mean.*

### Effect of Biostimulant Seed Priming on *Arabidopsis* Seed Germination

**FIGURE 3:**
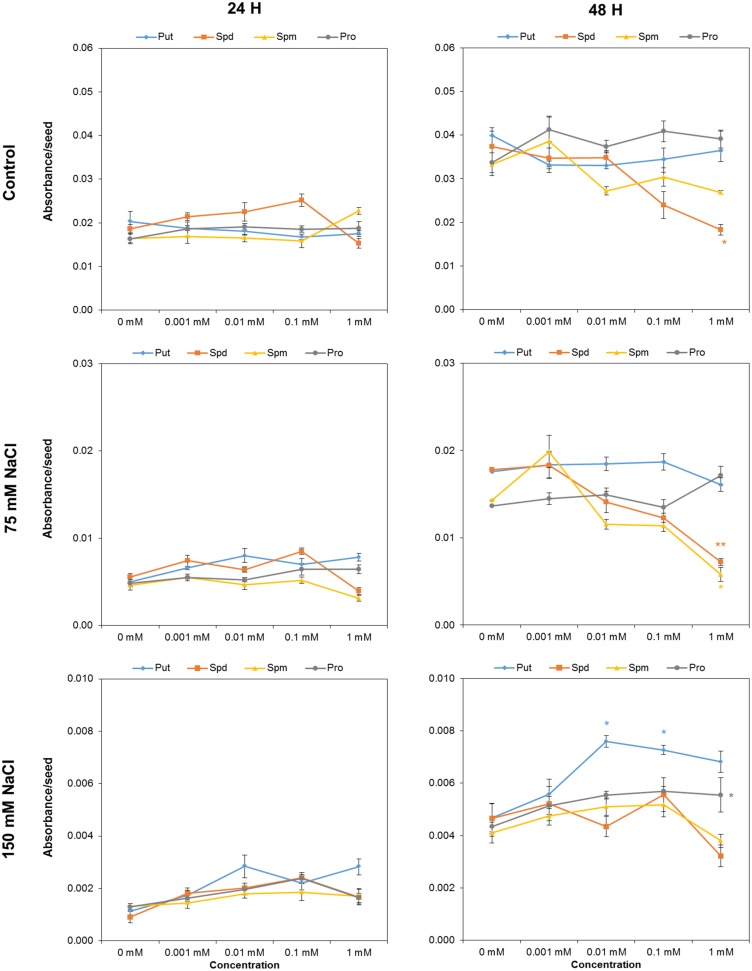
Absorbance per seed of *Arabidopsis* seeds primed with Put, Spd, Spm, and Pro at four concentrations (0.001, 0.01, 0.1, and 1 mM), after 24 or 48 h of germination. *Mean ± SE*. Statistical analysis was performed via the Kruskal-Wallis test. Asterisks indicate differences relative to the non-treated variant ^∗∗^*p* < 0.01; ^∗^*p* < 0.05.

We used the above-described optimized protocol to evaluate the effect of biostimulant (Put, Spd, Spm, and Pro) seed priming on seed germination under salt stress conditions. After 24 h, the tested variants differed only slightly (**Figure 3**). However, 1 mM Spd inhibited seed germination under control conditions and after 48 h of exposure to 75 mM NaCl, but exerted no effect under severe salt conditions (**Figure 3**). The same holds true for 1 mM Spm which also inhibited seed germination in 75 mM NaCl. The most visible effect was obtained for seeds primed with 0.01 and 0.1 mM Put and (to a lesser extent) 1 mM Pro, which yielded a significant increase in the germination in 150 mM NaCl (**Figure 3**).

### Seed Size Conditions Associated With *Arabidopsis* Rosette Growth

**FIGURE 4:**
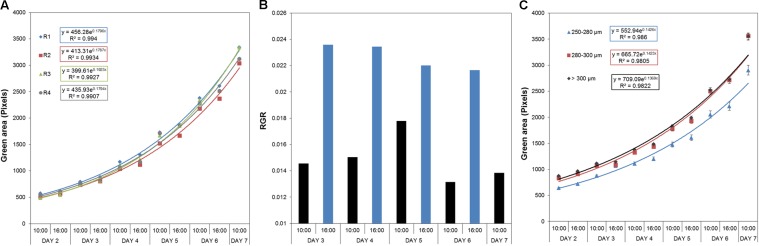
Natural variation in *Arabidopsis* rosette growth in 48 multi-well plates under control conditions. **(A)** Green area (pixels) associated with the growth of four DAG *Arabidopsis* seedlings in independent 48-well plates (replicates; R1–R4) for 7 days. *Mean ± SE.* **(B)** Relative growth ratio (RGR, pixel pixel^−1^ hour^−1^) of four DAG *Arabidopsis* seedlings grown in 48-well plates (*n* = 192). **(C)** Effect of the seed size on the green area (pixels) associated with the growth of four DAG *Arabidopsis* seedlings in independent 48-well plates. Three different size categories of seeds were considered: 250–280, 280–300, and >300 μm. The equation of the curve and the Pearson’s correlation coefficient were calculated. 250–280 μm seeds were significantly smaller than 280–300 and >300 μm ones, according to the multiple comparisons after ANOVA.

To determine the effect of biostimulants on the early seedling development of *Arabidopsis* plants under salt stress conditions, we further optimized our previously published protocol (De Diego et al., 2017) for HTS of the rosette growth. For rapid characterization of the plant biostimulants, the protocol was improved as follows: the response of 4-day-old *Arabidopsis* seedlings grown in 1× MS was evaluated using 48 well plates with four biological replicates randomly distributed in the platform. Due to the rapid image acquisition of our system (∼250 plates per hour) the seedlings were imaged twice per day (at 10:00 and at 16:00) for seven consecutive days (**Supplementary Figure S1**). The time-dependent increase in the rosette area (represented by the green region) and RGR were determined for each replicate. The green area differed negligibly among the replicates according to ANOVA (**Figure 4A**), which also exhibited similar RGR. Using this approach, we could record fluctuations in the RGR (per hour) between the 2 days sessions, thereby increasing the sensitivity and applicability of the assay to analysis of circadian rhythms. Higher RGR occurred in the period from 10:00 a.m. to 4:00 p.m. (**Figure 1B**) than in other sessions.

The effect of seed size on the variability of early seedling development via rosette growth was evaluated to further increase the technical precision of the assay. Using sieves, the seed batch was separated into three different size categories: 250–280, 280–300, and >300 μm. Seeds larger than 280 μm produced seedlings with similar rosette area (see **Figure 4C**), whereas seeds with sizes of 250–280 μm yielded significantly smaller rosettes (**Supplementary Table S1**). Although seeds with sizes of 280–300 μm were quite abundant, seeds larger than 300 μm were rare. Thus, due to their abundance and good growth performance, we selected the 280–300 μm seeds as the standard for subsequent experiments.

### HTS of *Arabidopsis* Rosette Growth as a Suitable Assay for the Characterization of Biostimulants Under Control and Salt Stress Conditions

**FIGURE 5:**
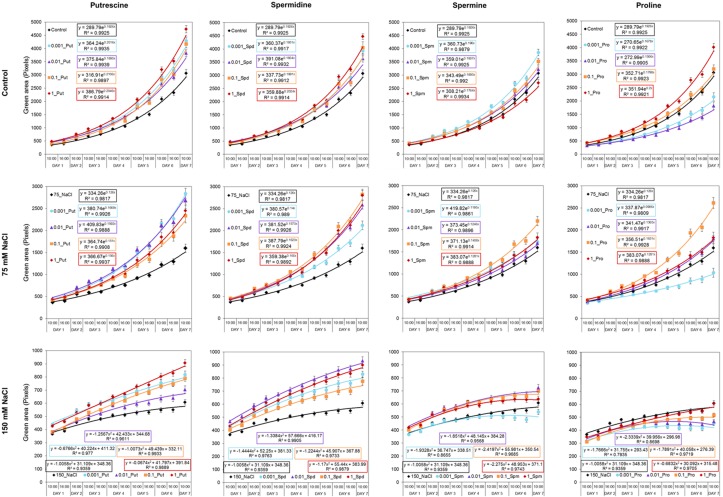
Variation in *Arabidopsis* rosette growth from primed seeds with moderate and severe salt stress. Green area (pixels) of four DAG *Arabidopsis* seedlings primed with Put, Spd, Spm, and Pro at four concentrations (0.001, 0.01, 0.1, and 1 mM) and grown for 7 days in 48-well plates under control, moderate (75 mM NaCl), and severe (150 mM NaCl) salt stress conditions. *Mean ± SE.* The equation of the curve and the Pearson’s correlation coefficient were calculated.

**FIGURE 6:**
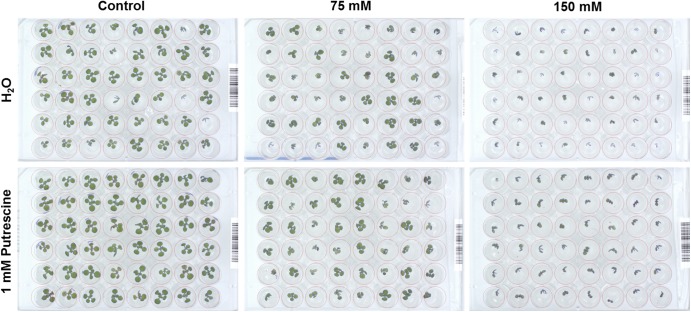
RGB image of an individual 48-well plate containing four DAG *Arabidopsis* seedlings primed with 1 mM Put grown for 7 days under control, moderate (75 mM NaCl), and severe (150 mM NaCl) salt stress conditions.

**FIGURE 7:**
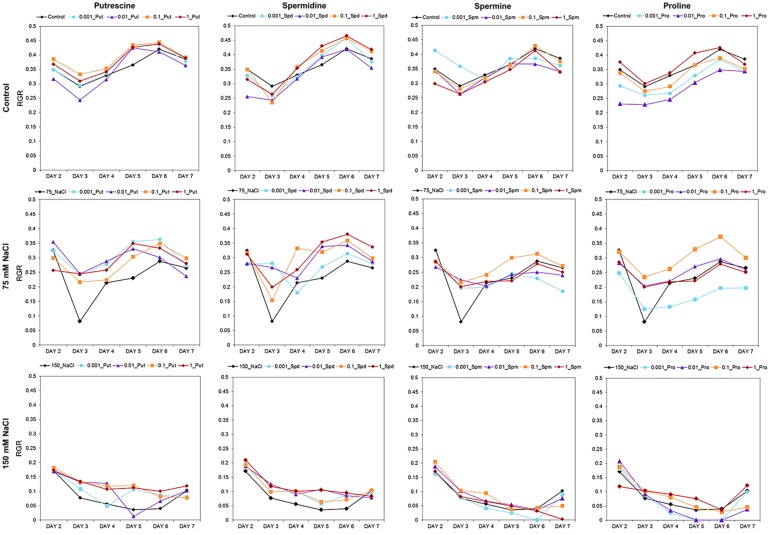
Relative growth ratio (RGR, pixel pixel^−1^ day^−1^) of four DAG *Arabidopsis* seedlings primed with Put, Spd, Spm, and Pro at four concentrations (0.001, 0.01, 0.1, and 1 mM) and grown for 7 days in 48-well plates under control, moderate (75 mM NaCl), and severe (150 mM NaCl) salt stress conditions.

Our OloPhen platform has sufficient capacity for the simultaneous testing of numerous variants (De Diego et al., 2017). To demonstrate the capacity for large-scale stress-response studies, we performed an experiment analogous to the germination assay using a 1× MS medium supplemented with two concentrations of NaCl (75 or 150 mM). The seeds were primed with Put, Spd, Spm, and Pro over the same concentration range (0.001, 0.01, 0.1, and 1 mM) described in the Methods section. The 4-day old seedlings were transferred for continued growth under three different conditions: control, moderate salt (75 mM NaCl) and severe salt (150 mM NaCl). In this experimental design, 119 units of 48 well plates containing a total of 5,712 plants were analyzed in a single run. As shown in **Figure 5**, seed priming with biostimulants induced significant differences in the rosette growth of individual variants (**Supplementary Table S2**). All concentrations of Put and Spd improved rosette growth and RGR, in both control and salt stress conditions, acting as plant growth promotors and stress alleviators (**Figure 5**). The best results were obtained with Put and Spd (**Figures 5**, **6**), especially under the severe salt condition (150 mM NaCl). In this case, exponential growth of the plants was maintained (**Figure 5**) through more efficient RGR per day (**Figure 7**) than that associated with other conditions. Spm priming promoted concentration-dependent growth under control and moderate salt stress conditions, although this growth stimulation was less than that induced by Put or Spd (**Figure 5**). Although Spm application improved rosette growth under severe stress conditions, maximum growth of the Spm-primed seedlings occurred earlier than that of seedlings grown only with 150 mM NaCl (**Figure 5**). Spm can therefore be classified as a plant growth promotor rather than a stress alleviator. In the case of stress-related amino acid Pro, we observed that low concentrations of Pro inhibited plant growth, whereas the highest concentrations stimulated growth in control and 75 mM NaCl conditions (**Figure 5** and **Supplementary Table S2**). Under the moderate stress induced by 75 mM NaCl, high concentrations of Pro exerted a stress-alleviating effect, but had a rather negative effect under the severe salt stress condition (**Figure 5** and **Supplementary Table S2**).

### Effect of Biostimulant on *Arabidopsis* Seedling Establishment

**FIGURE 8:**
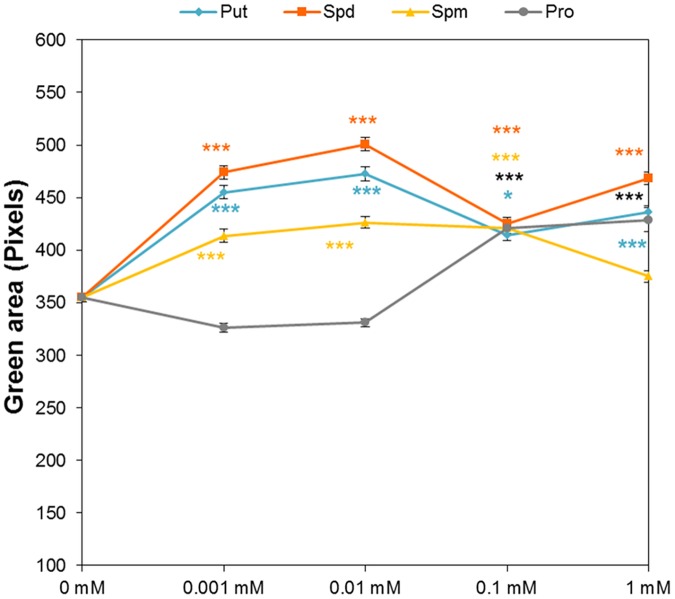
Effect of biostimulant seed priming on the seedling establishment of *Arabidopsis*. Green area (pixels) of four DAG *Arabidopsis* seedlings primed with Put, Spd, Spm, and Pro at four concentrations (0.001, 0.01, 0.1, and 1 mM) and grown under control conditions after the transfer to 48-well plates. Statistical analysis was performed via the Kruskal-Wallis test. Asterisks indicate differences relative to the non-treated variant. ^∗∗∗^*p* < 0.001; ^∗^*p* < 0.05.

Analysis of the dataset recorded from the above-described HTS of rosette growth revealed the effect of seed priming on early-seedling establishment. In this case, we analyzed the green area of the *Arabidopsis* seedlings immediately after the transfer to 48 well plates, corresponding to time zero of the HTS focused on *Arabidopsis* rosette growth as a suitable assay. Without salt stress, the sizes of seedlings established from primed seeds differed significantly from the sizes of seedlings resulting from non-primed seeds (**Figure 8**). For the entire range of concentrations, the priming by Put and Spd resulted in significantly larger rosettes compared to those seedlings from non-primed seeds. Except for the highest (1 mM) concentration, all Spm concentrations lead to a significant increase in the green area of the seedlings, whereas for Pro a considerable increase was observed only at the highest concentration (**Figure 8**). These results showed that our method can record traits in a complex manner that describes the effect of priming on all important stages of early development (e.g., germination, early seedling establishment and rosette growth).

### Effect of Biostimulants on the Leaf Color of *Arabidopsis* Rosettes Under Control and Salt Stress Conditions

**FIGURE 9:**
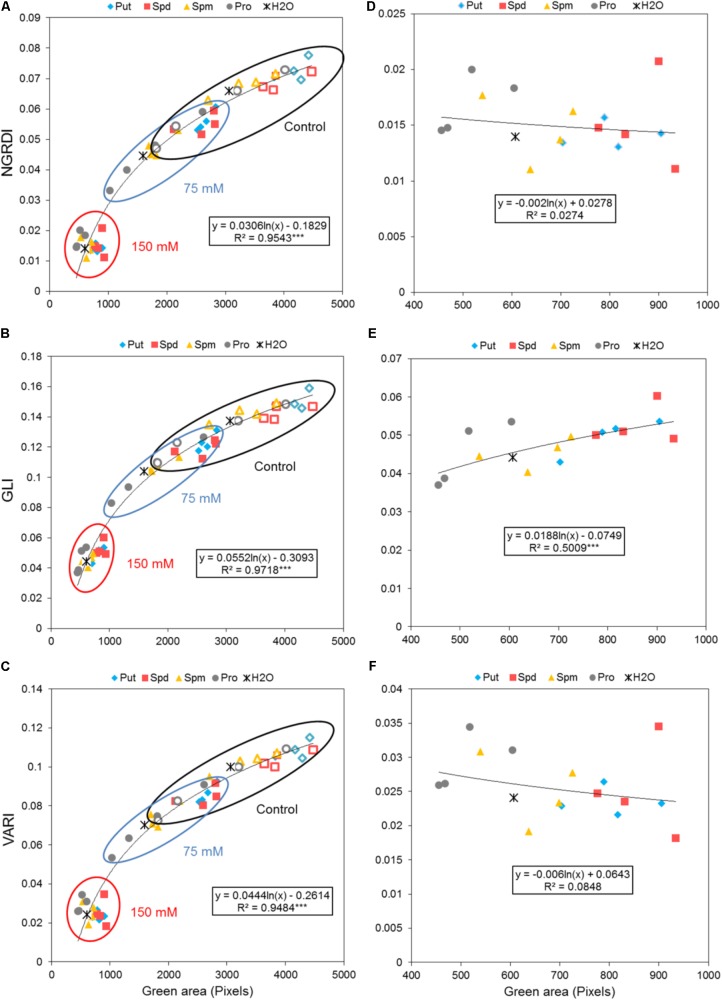
Effect of biostimulant seed priming on the stress response of *Arabidopsis*. **(A)** Correlation between the color index; NGRDI **(A)**, GLI **(B)**, or VARI **(C)** and the green area (Pixels) of four DAG *Arabidopsis* seedlings primed with Put, Spd, Spm, and Pro at four concentrations (0.001, 0.01, 0.1, and 1 mM) and grown under control (empty symbols), moderate (75 mM NaCl), and severe (150 mM NaCl) (filled symbols) salt stress conditions for 7 days. **(B)** Correlation between NGRDI **(D)**, GLI **(E)**, or VARI **(F)** and the green area of the *Arabidopsis* seedlings grown only under severe stress conditions. The equation of the curve and the Pearson’s correlation coefficient with significance, according to ANOVA, were calculated after linearization. ^∗∗∗^*p* < 0.001.

The degradation of chlorophyll, manifested as a change in leaf color, represents one of the most important symptoms of stresses in plants. This change in color may serve as an important marker in stress-related plant studies, especially in those employing salinity. To obtain this information, we introduced another trait into our method describing the effect of seed priming on the plant stress response. As described in the Methods section, the leaf color of the *Arabidopsis* rosettes was determined. We also evaluated the potential of three vegetation indices (VI) calculated using all three mixed visible bands (i.e., R, G, and B bands) which included the NGRDI, GLI, and VARI as indicators of leaf color change. These indices were strongly correlated with changes in the rosette area of the *Arabidopsis* seedlings and the values thereof depended on the seed priming treatment and salt intensity (**Figures 9A,B**). Of the three indices, GLI exhibited the highest sensitivity to salt stress (*R^2^*: 0.97; *R^2^* for NGRDI and VARI: 0.95). However, when the three VI were separately evaluated for the seedling with 150 mM NaCl, a significant positive correlation with the green area of the *Arabidopsis* rosette (**Figures 9D–F**) was obtained only for GLI. The seed priming with Put and Spd generated *Arabidopsis* rosettes with the highest greenness under control and salt stress conditions. The highest values were observed for GLI where 1 mM Put and Spd yielded 22 and 31%, respectively, higher levels of greenness than that of the non-treated seeds (**Figure 9E**).

### PBC Index for Estimating the Biostimulant Mode of Action

**FIGURE 10:**
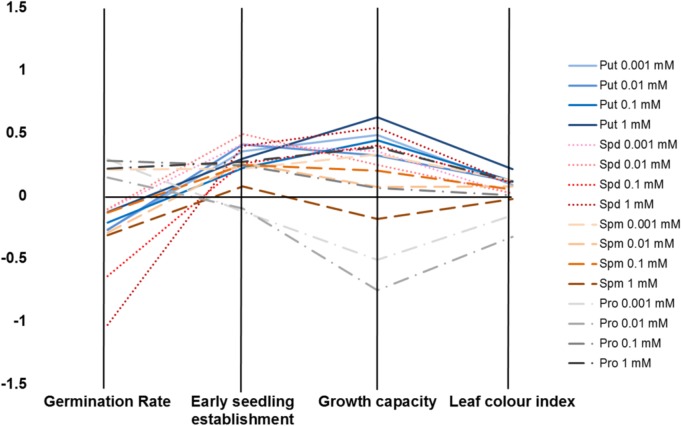
Characterization of plant biostimulants under control conditions. Parallel coordinate plot of the traits (germination, seedling establishment, plant growth capacity, and leaf color index) obtained from the Multi-trait HTS of *Arabidopsis* seeds primed with Put, Spd, Spm, and Pro at four concentrations (0.001, 0.01, 0.1, and 1 mM) and grown under control, moderate (75 mM NaCl), and severe (150 mM NaCl) salt stress conditions.

**FIGURE 11:**
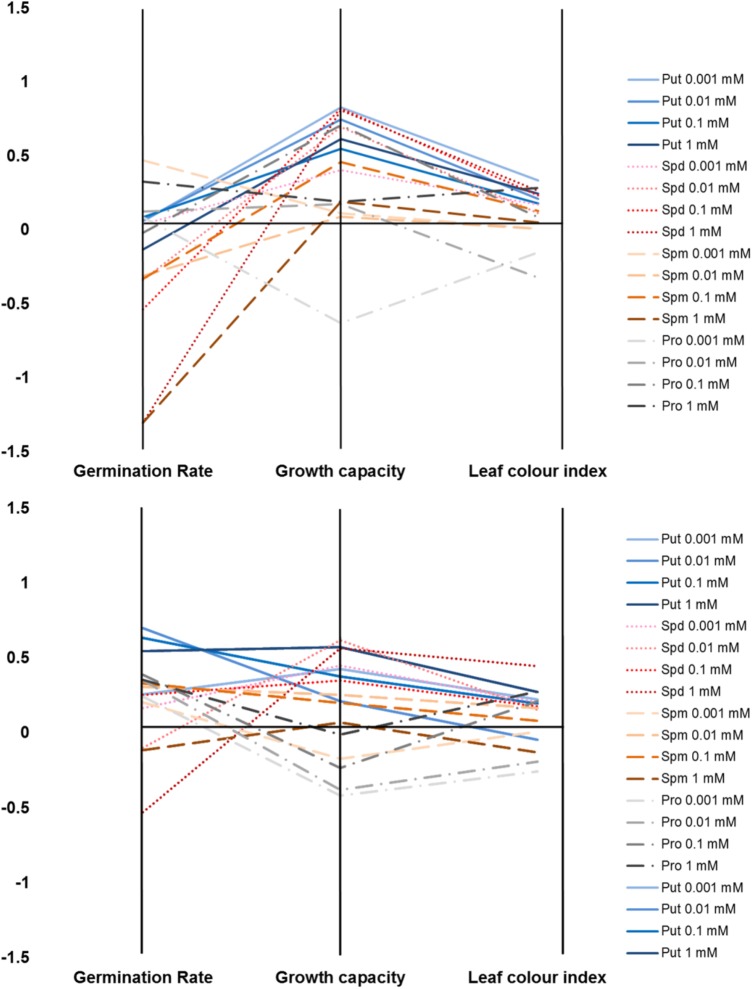
Characterization of plant biostimulants under moderate **(A)** or severe **(B)** salt stress conditions. Parallel coordinate plot of the traits (germination, plant growth capacity, and leaf color index) obtained from the Multi-trait HTS of *Arabidopsis* seeds primed with Put, Spd, Spm, and Pro at four concentrations (0.001, 0.01, 0.1, and 1 mM) and grown under moderate (75 mM NaCl) or severe (150 mM NaCl) salt stress conditions.

We developed a Plant Biostimulant Characterization (PBC) index aimed at integrating both HTS methods into a pipe-line that yields straight-forward information allowing simple selection of the best treatment under each condition. The index can represent up to four analyzed traits: seed germination rate (%), seedling establishment (green pixels after transfer to 48 well plates), growth capacity (Pixels) and the leaf color index (GLI) for the primed and non-primed seeds. For the index calculation first the differences between the controls of the different growth conditions and variants (compound and concentration) under the same conditions were calculated as the log2 of the ratio. The number represented by the independent traits and treatment constituting the PBC index can be then represented in a parallel coordinate plot (**Figures 10**, **11**). This type of representation allows a better visualization (than that provided by other representations) of the variant-induced changes in each trait. In addition, the connection between the traits can be quickly identified. For example, under control conditions, it is easier to visualize that the seed priming with Put and Spd mainly improved *Arabidopsis* growth capacity, and to less extend the early seedling establishment and leaf color index, whereas the germination remained unchanged or was even inhibited by these agents (**Figure 10**). Under salt stress condition, seed priming with polyamines improved *Arabidopsis* growth capacity and leaf color index under both intensities tested (75 and 150 mM) (**Figures 11A,B**). Nevertheless, only under severe conditions, the priming with polyamines improved seed germination in almost all cases compared with their respective control (**Figure 11A**).

**FIGURE 12:**
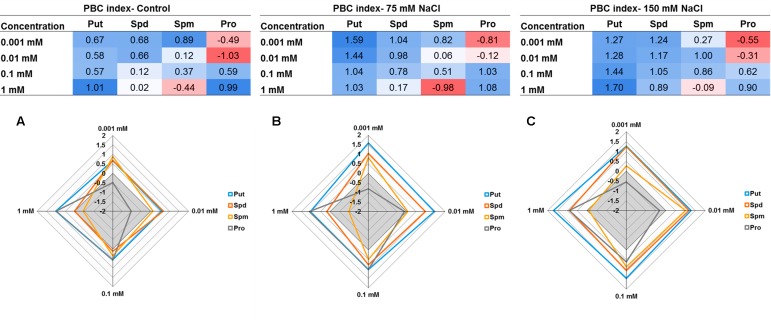
Tables and Radar chart of the Plant Biostimulant Characterization (PBC) index by summing the relative changes (log2) obtained for the parallel coordinate plot for each compound (Put, Spd, Spm, or Pro) concentration (0.001, 0.01, 0.1, and 1 mM) and growth condition; control **(A)**, 75 mM NaCl **(B)**, or 150 mM NaCl **(C)**.

The concentration effect of the tested compound under three different growth conditions (control, 75 mM NaCl or 150 mM NaCl) was then determined by summing the relative changes (log2) obtained for the parallel coordinate plot ending with a single number as shown in **Figure 12**. This sum yielded a total that can reach a positive (biostimulant- blue) or negative (inhibitor-red) value. The resulting numbers were then plotted in a multidimensional graphic “radar chart” using the concentrations as quantitative variables (**Figure 12**). From these results we confirmed that Put was the most efficient plant growth promotor and stress alleviator with higher values in each concentration and growth condition, compared with the controls. The remaining compounds exhibited a concentration- and growth-condition-dependent response. For example, Spd and Spm yielded the highest index values at low concentrations, whereas Pro acted as plant biostimulants at high concentrations only, and its effectiveness increased with increasing salt stress intensity (**Figures 12B,C**). These results confirm that the presented MTHTS approach is an adequate tool for a fast and simultaneous analysis of various concentrations and growth conditions for identification and, especially, characterization of the operation mode associated with new biostimulants.

## Discussion

Uniform and efficient seed germination and establishment of early seedlings are crucial for agricultural crop production under stress conditions, especially drought and/or salinity (Savvides et al., 2016). Seed priming, where seeds are pre-sown with certain compounds with the aim of increasing the uniformity and vigor of seedlings, represents an innovative alternative to coping with the negative stress effects. In addition, the use of natural compounds or biostimulants as priming agents can improve the efficiency of crop production and yield under suboptimal conditions. The use of these substances is more sustainable and environmentally friendly compared with the use of other materials. The priming with single compounds such as polyamines and amino acids can be a good technology against different abiotic stresses (Savvides et al., 2016). However, despite the fact that most of the complex biostimulants of several origins (i.e., protein hydrolysis from agroindustrial by-product from both plant sources and animal waste, and seaweed extracts) contain these types of compounds (du Jardin, 2015), their biostimulant activity potential hasn’t been fully evaluated. For this reason, we used in this study the stress related amino acid Pro and polyamines’ representatives as priming agents to bring additional information about their possible biostimulant mode of action. Therefore, biostimulant manufacturers require tools for identifying new biostimulants, characterizing and quantifying their biological effects and describing the corresponding mode of action. Moreover, during biostimulant preparation, the tools for rapid control of the quality during the extraction processes and production of different batches are needed. Taking into account the mentioned facts, we suggest that Put, Spd, Spm, and Pro have potential to be used as positive controls in the biostimulant research and manufacturing.

Screening platforms based on the semi-automated or automated bioassaying of simple traits based on *in vitro* *Arabidopsis* assays might be useful to accelerate the process for preliminary screening of stability, composition and effect of raw material. This testing allows for a rapid first-step screening on plants, eliminating the influence of soil and other environmental parameters (Povero et al., 2016). The testing of biostimulants using bioassays has been traditionally performed with Petri dishes, thus having low-throughput requiring posterior manual quantification (Durand et al., 2003; Colla et al., 2014; Povero et al., 2016). Recently, Rodriguez-Furlán et al. (2016) published an *in vitro* bioassay using *Arabidopsis* for the testing of several compounds. However, the use of scanners for image analysis yields an analysis rate of 20 min per plate and the analysis is performed only at one time-point (Rodriguez-Furlán et al., 2016). Several other manual and semi-automated HTS protocols using RGB imaging for phenotyping of *Arabidopsis* in the controlled conditions have been published with different throughputs and (dis)advantages. The method of Granier et al. (2006) showed possibilities solving potential complications and methodological difficulties with the spatial and temporal variability of micrometeorological conditions within a growth chamber, reaching throughput of 500 plants per hour. Recently, simple HTS protocol based on *in vitro* growth of *Arabidopsis* using square plates with 16 seedlings and manual image acquisition followed by analysis of plant size and color was published by Faragó et al. (2018). The protocol presented by us is based on our previous report of an automated method for HTS of *Arabidopsis* rosette growth in multi-well plates accessible at OloPhen facility (De Diego et al., 2017). The potential of this method was in our recent protocol improved in several ways through (1) increase of the number of plates per run from 480 to 572; (2) significant increase of the total number of plants analyzed by use of 48-well plates, instead of 24-well plates that increased the number of analyzed plants to more than 27.000 in less than 3 h; and (3) through increase of the resolution of the growth analysis by automated measurement twice a day within 1 week. As presented here, our new method allows a simultaneous study of different growth conditions without compromising the number of variants, replicates and plants per treatment. Moreover, compared to Faragó et al. (2018), the growth analysis of each plant is done for the whole cycle by imaging of the same plant individual. Further, the use of independent wells per plant permits an easier detection of the single plant so they are located in a concrete XY position. Thus, there is no requirement of any manual adjustment to separate individual plants. As clear example illustrating the potential of our method, in this work we automatically recorded the rosette growth of 5,712 *Arabidopsis* (119 plates × 48 seedling). The imaging of each well-plate was performed twice per day (at 10 a.m. and 4 p.m.) for 7 days, ending with 14 data points per plant in very short time. Altogether, we developed a very fast *in vitro* bioassay to analyze simultaneously a huge amount of treatments and plants.

The improved HTS of rosette growth under control and stress salinity was integrated in a pipe-line for the screening of biostimulants together with the HTS of *Arabidopsis* seed germination. For that, we developed a simple and fast bioassay for *Arabidopsis* seed germination based on (Pouvreau et al., 2013) using spectrophotometric analysis of MTT reduction in microtiter plates. With the classical method using a microscope, the distinction between non-germinated seeds and germinated seeds with a very short protruded radicle is very difficult, increasing the risk of germination rate underestimation (Pouvreau et al., 2013). However, the MTT method is simple and accurate and can be easily adapted for high-throughput germination bioassays. The HTS method is performed in 96 well plates. These plates allow many variants per plate (compounds, concentrations, and/or germination conditions) using a spectrophotometric MTT method with a simple read out of the germination rate per variant (**Figure 1**). In addition, we developed a simple in-house software routine to automatically count the seed number per well. This reduced the time consuming counting of the seed number per well necessary for increasing the accuracy of the method by reducing the variability within treatments (**Figure 2A**). Although free software applications exist for image-based analysis of seeds allowing automated definition of the seed shape and size (Tanabata et al., 2012), for our purpose we created a very simple software routine in MATLAB suitable for detecting and counting objects (seeds) in multi-well plates at 0 h (immediately after cold stratification and before seed germination). The obtained number is then used to recalculate the total absorbance of the well recorded by spectrophotometer to the absorbance per seed that represents the germination rate. This trait together with those obtained from the HTS of Arabidopsis rosette growth (plant establishment, plant growth capacity under different conditions and leaf color index), constitute the MTHTS for biostimulant characterization achievable within 1 week.

Many biostimulants contain various groups of components including complex mixtures of biologically active compounds and, hence, the testing should be performed over a broad concentration range allowing evaluation of concentration-dependent effects. We selected individual molecules as a first step in optimizing our bioassays for biostimulant characterization. The polyamines Put, Spd, and Spm, and the amino acid Pro, which also have been identified in the raw material of complex formulations from different natural origins, were selected (Colla et al., 2014; du Jardin, 2015). Moreover, we selected salinity as a stressor, owing to its negative impact on seed germination and plant growth. Using our approach, each compound can be simultaneously tested at different concentrations and plant growth conditions in both HTS methods. The results revealed differences in the mode of action for the four compounds applied to *Arabidopsis* seed germination and rosette growth (**Figures 3**–**9**). Put and Spd were identified as plant growth promotors and stress alleviators, whereas Spm and Pro were less efficient and their positive effect was concentration dependent (**Figures 5**, **10**, and **11**). The exogenous application of polyamines yields improved salt tolerance in many crops via enhanced germination and/or plant productivity (Roychoudhury et al., 2011; Li et al., 2015; Shekari et al., 2015). For example, exogenous application of Spd in *Cucumis sativus* L. induces accumulation of endogenous polyamines that act as free radical scavengers, thereby stabilizing cellular membranes and maintaining cellular ionic balance under salinity (Shu et al., 2012). This was attributed to a relatively high Put/(Spd+Spd) ratio that rendered seed priming with Put the most efficient treatment. As confirmation, Shu et al. (2015) demonstrated that Put application regulates protein expression at transcriptional and translational levels by increasing endogenous polyamine levels in thylakoid membranes which may stabilize the photosynthetic apparatus under a salt stress. In addition, changes in polyamines biosynthesis and catabolism influence plant tolerance and recovery capacity though a sophisticated crosstalk with plant hormones, which induces changes in primary metabolism such as the synthesis of amino acids, and improves photosynthesis and nutrient uptake under stress conditions (review in Podlešáková et al., 2018). Therefore, priming with polyamines could be a cheap, healthy, and easy solution for mitigating adverse salinity-induced stresses occurring during the initial developmental phases of crops.

The priming with Pro was less effective than with polyamines, and the most positive effect was in the germination rate under a severe salt stress. This may have resulted from the fact that enhanced Pro levels in plants occur in the first phases of seed germination and the seed-to-seedling transition (Silva et al., 2017). Similar results were obtained in rice, where the seeds pre-treated with Pro provided significant evidence for assessing the salt tolerance at the germination stage (Deivanai et al., 2011). However, the effect was variety dependent. In sugar cane grown *in vitro*, the anti-stress effect was also genotype dependent (Medeiros et al., 2015), but both dependences increased the stress tolerance by activating the plant antioxidative response. Other studies consider the Pro mode of action to be long-term, when the plant accumulated high levels of Pro, and attributed this action to plant recovery and hardening (De Diego et al., 2015; Sabagh et al., 2015). This could be explained by the fact that stress-tolerance improvement in many other crops required relatively high concentrations (Talat et al., 2013; Dawood et al., 2014). However, contradictory results regarding the Pro effect have been obtained for the same crop under the same stress conditions. For example, Teh et al. (2016) reported that 5 or 10 mM Pro improved salt stress tolerance of rice, but Deivanai et al. (2011) considered the 10 mM concentration toxic. This contradiction resulted mainly from the different intensities of salinity considered. Therefore, integrating a wide range of concentrations in the same bioassay combined with different stress levels for the testing of biostimulants constitutes a viable strategy for biostimulant mode of action characterization.

## Conclusion

In this work we present a complex pipe-line for a fast characterization of plant biostimulants suitable for seed-priming application giving straight-forward information for simple selection of the best treatments under control, moderate and severe salt stress conditions, using treatment evaluation through newly introduced index. The MTHTS approach based on the semi-automated analysis of *Arabidopsis* germination and rosette growth analyses four traits: *in vitro* germination rate, early seedling establishment capacity, growth capacity under stress and stress response based on plant greenness. The approach allows the acceleration of the biostimulant characterization through a simultaneous spanning of a broad number of biostimulants in a wide range of concentrations and stress conditions. Further, the method helps to define a biostimulant made of action based on its contribution to the plant development and stress tolerance such as plant growth promotor/inhibitor and/or stress alleviator. The presented approach (i) represents a useful tool for biostimulant research and development, and (ii) when combined with chemical-composition analysis and biological-activity measurements can help to identify the specific mode of action characterizing the biostimulants and their main bioactive ingredients.

## Author Contributions

LU, AH, JH, KD, LS, and NDD designed the experiments. LU, AH, JH, and KP performed the experiments. NDD and LS supervised the study and formulated the concept of the project. LU, AH, and NDD performed the data analysis. All authors discussed the results. LU, AH, JH, NDD, and LS wrote the manuscript.

## Conflict of Interest Statement

The authors declare that the research was conducted in the absence of any commercial or financial relationships that could be construed as a potential conflict of interest.

## Footnotes

1 http://www.plant-phenotyping.org/db_infrastructure#/tool/57

## Supplementary Material

FIGURE S1 *Arabidopsis* rosette growth in 48 multi-well plates for 7 days under control conditions.

TABLE S1 Statistical differences in the green area (pixels) of 4 DAG *Arabidopsis* seedlings from three different size categories of seeds (250–280, 280–300, and >300 μm) grown in 48-well plates (three biological replicates per treatment) for 7 days. Different letters indicate significant differences according to multiple comparisons performed after ANOVA.

TABLE S2 Statistical differences in the green area (pixels) of 4 DAG *Arabidopsis* seedlings primed with Put, Spd, Spm, and Pro at four concentrations (0.001, 0.01, 0.1, and 1 mM) and grown under control, moderate (75 mM NaCl) and severe (150 mM NaCl) salt stress conditions for 7 days. Different letters indicate significant differences according to multiple comparisons performed after ANOVA.

## Abbreviations

GLI: green leaf index
MTHTS: multi-trait high-throughput screening
NGRDI: normalized green red difference index
Pro: *L*-proline
Put: putrescine
Spd: spermidine
Spm: Spermine
VARI: visible atmospherically resistant index.
