# Supplementary figures and images for: Characterization of Biostimulant Mode of Action Using Novel Multi-Trait High-Throughput Screening of Arabidopsis Germination and Rosette Growth
Source: Front Plant Sci. 2018 Sep 13;9:1327. doi: 10.3389/fpls.2018.01327 (PMC6146039; doi:10.3389/fpls.2018.01327)

## Slide 1
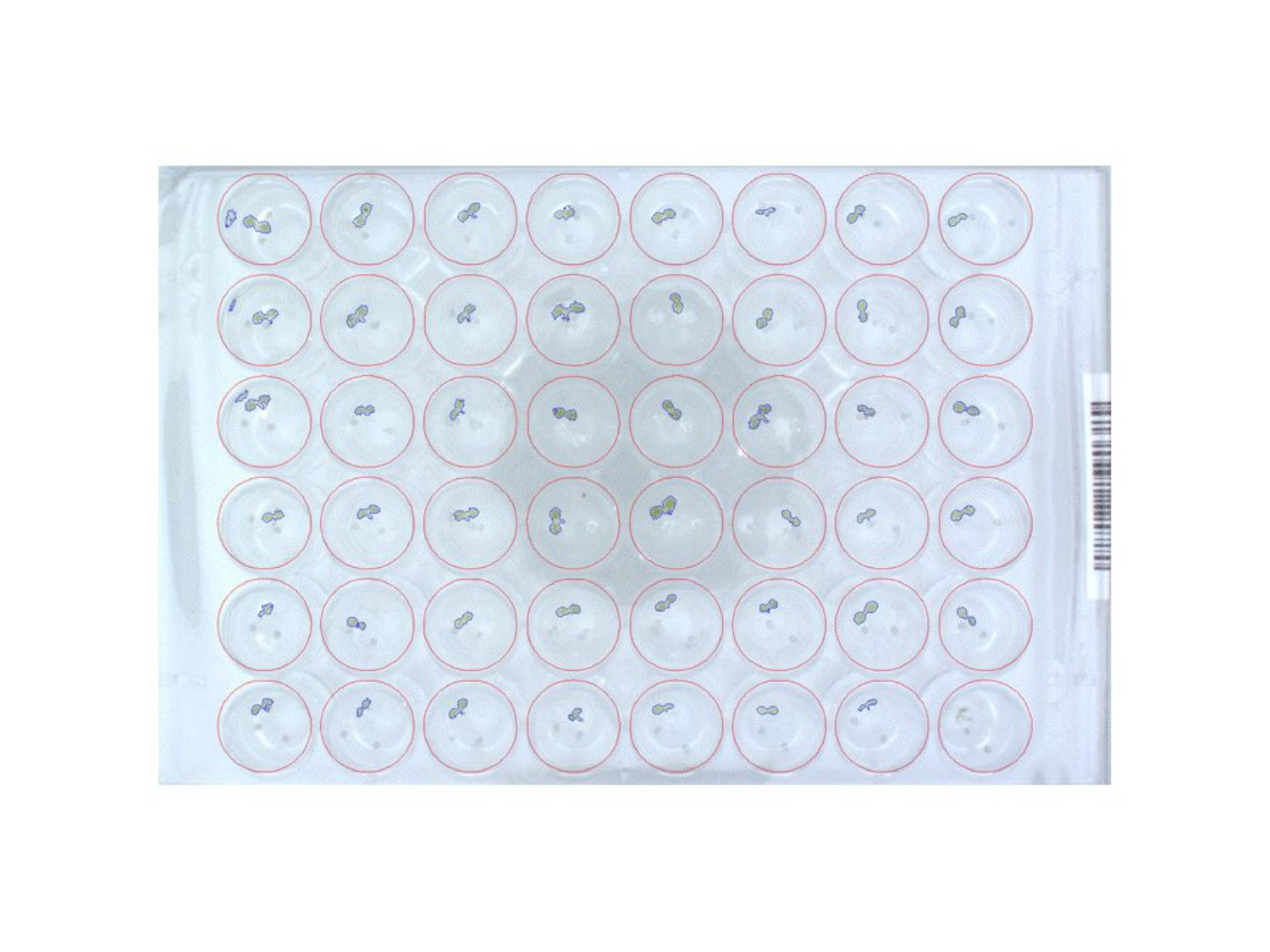

Supplement: FIGURE S1 — Arabidopsis rosette growth in 48 multi-well plates for 7 days under control conditions. [file Presentation_1.PPTX]
